# Analysis of a New Delayed HBV Model with Exposed State and Immune Response to Infected Cells and Viruses

**DOI:** 10.1155/2017/7805675

**Published:** 2017-11-16

**Authors:** Deshun Sun, Fei Liu

**Affiliations:** Control and Simulation Center, Harbin Institute of Technology, Harbin 15008, China

## Abstract

We propose a comprehensive delayed HBV model, which not only considers the immune response to both infected cells and viruses and a time delay for the immune system to clear viruses but also incorporates an exposed state and the proliferation of hepatocytes. We prove the positivity and boundedness of solutions and analyze the global stability of two boundary equilibria and then study the local asymptotic stability and Hopf bifurcation of the positive (infection) equilibrium and also the stability of the bifurcating periodic solutions. Moreover, we illustrate how the factors such as the time delay, the immune response to infected cells and viruses, and the proliferation of hepatocytes affect the dynamics of the model by numerical simulation.

## 1. Introduction

Hepatitis B virus (HBV) has become one of the serious infectious diseases threatening global human health, which can cause chronic liver infection and further result in liver inflammation, fibrosis, cirrhosis, or even cancer [1]. Each year more than 1 million people die of end-stage liver diseases like cancer due to the HBV infection [2].

Mathematical modeling and analysis of the dynamics of such infectious viruses as HBV play important roles in understanding the factors that govern the infectious disease progression and offering insights into developing treatment strategies and guiding antiviral drug therapies [3]. So far, there have been plenty of mathematical models proposed to describe and analyze virus infection, immune responses, and antiretroviral treatment [4–10].

Among these works, the development of virus models with immune responses is gaining much attention [3, 11, 12]. The immune system is essential in controlling the level of virus reproduction in terms of the strength of the Cytotoxic T Lymphocyte (CTL) response. A small change of the CTL response may have a large effect on virus production and infected cells load. As to this aspect, typical work can be summarized as follows. Chen et al. [12] indicated that the immunity system can not only clear free viruses but also kill infected cells. Elaiw and AlShamrani [3] proposed a four-dimensional model with humoral immunity response and general function and analyzed the global asymptotic stability of all equilibria based on the general function. However, both models in [3, 12] do not consider time delay. In order to characterize the time of a body's immune response after the virus infection of target cells, time delay has been taken into account [13–16]. For example, Zhu et al. [16] proposed an HIV infection model with CTL response delay and analyzed the effect of time delay on the stability of equilibria. Besides, a latent period would be necessary to be incorporated into a virus model because when viruses infect a healthy organ like liver, it will not be pathogenetic at once, as it takes about six weeks to six months from the infection to the incidence [17–20]. For example, Medley et al. [17] proposed an HBV model with an exposed state, namely, infected but not yet infectious. Moreover, it was modeled in [21, 22] that the liver can regenerate cells and compensate the lost infected hepatocytes by the proliferation of hepatocytes.

In this paper, we will propose a more comprehensive model than those existing ones, which not only considers the immune response to both infected cells and viruses and a time delay for the immune system to clear viruses but also incorporates an exposed state and the proliferation of hepatocytes. We first discuss the existence of two boundary equilibria and one positive (infection) equilibrium. We then analyze the global stability of the two boundary equilibria, the local asymptotic stability and Hopf bifurcation of the positive equilibrium and also the stability of the bifurcating periodic solutions. Moreover, we perform numerical simulations to illustrate some of the theoretical results we obtain and also illustrate how the factors such as the immune response to infected cells and viruses and the proliferation of hepatocytes affect the dynamics of the model under time delay.

The paper is structured as follows. In Section 2, a delayed mathematical model is proposed, and the positivity and boundedness of solutions, existence of two boundary equilibria, and one positive equilibrium are discussed, followed by the global stability analysis of these two boundary equilibria and the local asymptotic stability and Hopf bifurcation of the positive equilibrium in Section 3. The stability of the bifurcating periodic solutions is studied in Section 4. In Section 5, some numerical simulations and discussions are given. Finally, a conclusion is given in Section 6.

## 2. Mathematical Model

 Wang et al. [23] proposed a virus infection model of four-dimensional equations with delayed humoral immune response, which, however, does not involve an exposed state and consider the proliferation of hepatocytes. Although it considers the immune response to viruses, it does not involve the immune response to infected cells.

**Figure 1:**
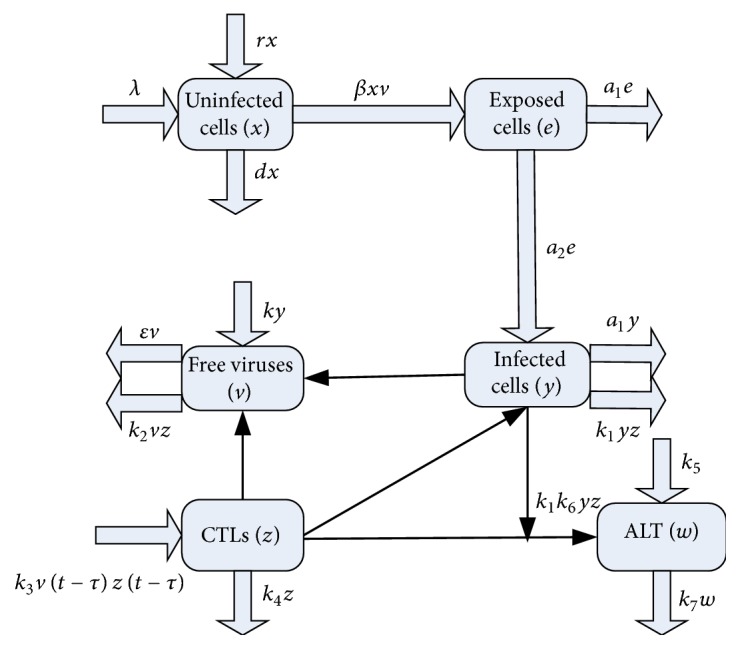
The mechanism of our model.

Based on this model, we propose a new and comprehensive HBV model, which not only considers the immune response to both infected cells and viruses and a time delay for the immune system to clear viruses but also incorporates an exposed state and the proliferation of hepatocytes. To better understand our model, we illustrate its mechanism in Figure 1.

The model is then given as follows:

(1) x˙t=λ+rxt−dxt−βxtvt,e˙t=βxtvt−a1et−a2et,y˙t=a2et−a1yt−k1ytzt,v˙t=kyt−εvt−k2vtzt,z˙t=k3vt−τzt−τ−k4zt,w˙t=k5+k1k6ytzt−k7wt,

where *x*,  *e*,  *y*,  *v*,  *z*, and *w* denote the number of uninfected cells, exposed cells, infected cells, free viruses, CTLs, and alanine aminotransferases (ALT), respectively. The parameter *λ* represents the natural production rate of uninfected cells. *rx* is a new term which is introduced to represent the proliferation of hepatocytes, where *r* is the proliferation rate. Parameters *d*, (and the following) *a*_1_,  *ε*,  *k*_4_, and *k*_7_ represent the natural death rate of uninfected cells, exposed cells, infected cells, free viruses, CTLs, and ALT, respectively. *β* represents the infection rate from uninfected cells to exposed cells and *a*_2_ the transfer rate from exposed cells to infected cells. The production rate of free viruses from infected cells is denoted by *k*, and the production rate of CTLs by *k*_3_. *k*_5_ represents the production rate of ALT from the extrahepatic tissue and *k*_1_ *k*_6_ the production rate of ALT when the infected hepatocytes are killed by CTL. The immunity-induced clearance for infected cells is modeled by a term *k*_1_ *yz*, where *k*_1_ represents the clearance rate of infected cells. Similarly, the immunity-induced clearance for free viruses is modeled by *k*_2_ *vz*, where *k*_2_ represents the clearance rate of free viruses. *τ* is time delay. All the parameters in this paper are positive and *d* > *r*. For convenience, we define new parameter *ρ* = *d* − *r*.

### 2.1. Positivity and Boundedness of Solutions

In this subsection, we prove the positivity and the boundedness of solutions of system (1).

We denote *x*(0) ≥ 0,  *e*(0) ≥ 0,  *y*(0) ≥ 0,  *w*(0) ≥ 0,  *v*(*t*) ≥ 0,  *z*(*t*) ≥ 0,  *t* ∈ [−*τ*, 0]. From the first equation of system (1), we have *x*(*t*) = *e* ^−∫_0_^*t*^(*ρ*+*βv*(*s*))*ds*^ *x*(0) + *λ*∫_0_^*t*^ *e* ^−∫_*s*_^*t*^[*ρ*+*βv*(*ξ*)]*dξ*^ *ds*; therefore, *x*(*t*) ≥ 0 for ∀*t* > 0 if *x*(0) ≥ 0. Next, we consider the second, third, and fourth equation in system (1) as a nonautonomous system for *e*(*t*), *y*(*t*), *v*(*t*):

(2) e˙=βxv−a1+a2et,y˙=a2e−a1+k1zyt,v˙=ky−ε+k2zvt.

Based on Theorem 2.1 in [24], we have *e*(*t*) ≥ 0,  *y*(*t*) ≥ 0,  *v*(*t*) ≥ 0 if *e*(0) ≥ 0,  *y*(0) ≥ 0,  *z*(0) ≥ 0.

*z*(*t*) = *e* ^−*k*_4_*t*^ *y*(0) + ∫_0_^*t*^ *k*_3_ *v*(*t* − *γ*)*z*(*t* − *γ*)*e* ^−*k*_4_(*t*−*γ*)^ *dγ*, we have *z*(*t*) ≥ 0,  ∀*t* > 0 if *z*(*t*) ≥ 0,  *t* ∈ [−*τ*, 0].

*w*(*t*) = *e* ^−*k*_7_*t*^ *w*(0) + *e* ^−*k*_7_*t*^∫_0_^*t*^[(*k*_5_ + *k*_1_ *k*_6_ *y*(*s*)*z*(*s*)]*e* ^−*k*_7_*t*^ *ds*, because *y*(*t*), *z*(*t*) ≥ 0, so we have *w*(*t*) ≥ 0,  ∀*t* > 0 if *w*(0) ≥ 0.

Hence, the nonnegative is proved. In what follows, we will study the boundedness of solutions. We define *G*(*t*) as a linear combination of *x*,  *e*,  *y*,  *v*,  *z*:

(3) Gt=xt+et+yt+a12kvt+a1k22kk3zt+τ,δ=mind−r,a12,a1,ε,k4,dGtdt=λ−ρx−βxv+βxv−a1e−a2e+a2e−a1y−k1yz+a12kky−εv−k2vz+a1k22kk3k3vtzt−k4zt+τ=λ−d−rx−a12e−a1y−a1ε2kv−a1k2k42kk3zt+τ−k1yz≤λ−δGt.

Therefore, we obtain lim_*t*→*∞*_⁡*G*(*t*) ≤ *λ*/*δ*, namely, *x*(*t*) + *e*(*t*) + *y*(*t*) + (*a*_1_/2*k*)*v*(*t*) + (*a*_1_ *k*_2_/2*kk*_3_)*z*(*t* + *τ*) ≤ *λ*/*δ*. So we have 0 ≤ *x*(*t*), *e*(*t*), *y*(*t*), *v*(*t*), *z*(*t*) ≤ *λ*/*δ* Because the boundedness of *x*(*t*), *e*(*t*), *y*(*t*), *v*(*t*), *z*(*t*),  lim_*t*→*∞*_⁡*w*(*t*) ≤ (*k*_5_ + *k*_1_ *k*_6_)*λ* ^2^/*k*_7_ *δ* ^2^.

The boundedness is proved.

### 2.2. Equilibrium

In this subsection, we study the equilibria of system (1). The method to obtain equilibria is setting $\dot{x}=\dot{e}=\dot{y}=\dot{v}=\dot{z}=\dot{w}=0$ and computes the following:

(4) λ−ρx−βxv=0,βxv−a1e−a2e=0,a2e−a1y−k1yz=0,ky−εv−k2vz=0,k3vt−τzt−τ−k4z=0,k5+k1k6yz−k7w=0.

The system (1) has two boundary equilibria (an infection-free equilibrium *E*_00_ in which *x* ≠ 0,  *w* ≠ 0,  *e* = *y* = *v* = *z* = 0 and an equilibrium without immune response *E*_11_ in which *x* ≠ 0,  *e* ≠ 0,  *y* ≠ 0,  *v* ≠ 0,  *w* ≠ 0,  *z* = 0) and a positive (infection) equilibrium *E*_22_ in which *x* ≠ 0,  *E* ≠ 0,  *y* ≠ 0,  *v* ≠ 0,  *z* ≠ 0,  *w* ≠ 0.

The infection-free equilibrium is *E*_00_ = (*x*_0_, 0,0, 0,0, *w*_0_), where *x*_0_ = *λ*/*ρ*, and *w*_0_ = *k*_5_/*k*_7_, and the basic reproductive number is obtained by the following method.

Based on integral operator spectral radius, the basic reproductive number is *R*_0_ = *ρ*(*FV* ^−1^), where

(5) F=00βx0a2000k0,V=a1+a2000a1+k1z0000ε.

Hence, we have the basic reproductive number being *R*_0_ = *a*_2_ *kx*_0_ *β*/*a*_1_ *ε*(*a*_1_ + *a*_2_).

The equilibrium without immune response is *E*_11_ = (*x*_1_, *e*_1_, *y*_1_, *v*_1_, 0, *w*_1_), where *x*_1_ = *a*_1_ *ε*(*a*_1_ + *a*_2_)/*a*_2_ *kβ*,  *e*_1_ = (*a*_1_ *ε*/*a*_2_ *k*)*v*_1_,  *y*_1_ = (*ε*/*k*)*v*_1_,  *v*_1_ = *a*_2_ *kλ*/*a*_1_ *ε*(*a*_1_ + *a*_2_) − *ρ*/*β*, and *w*_1_ = *k*_5_/*k*_7_. Similarly, we have the basic reproductive number is *R*_1_ = *k*_3_ *v*_1_/*k*_4_ + *kk*_3_ *y*_1_ *k*_1_/*a*_1_ *k*_2_ *k*_4_ at *E*_11_ = (*x*_1_, *e*_1_, *y*_1_, *v*_1_, 0, *w*_1_).

The infected positive equilibrium is *E*_22_ = (*x*_2_, *e*_2_, *y*_2_, *v*_2_, *z*_2_, *w*_2_), where

(6) v2=k4k3,x2=λρ+βv2,e2=βx2v2a1+a2,y2=A+A2+4B2,A=a1k2k4−k1k3εv2kk1k3,B=a2k2k4kk1k3>0,z2=k3ky2−εv2k2k4,w2=k5+k1k6y2z2k7.

## 3. Analysis

### 3.1. Global Stability Analysis of the Two Boundary Equilibria

In this section, we will employ the direct Lyapunov method and LaSalle's invariance principle to establish the global asymptotic stability of the two boundary equilibria.

**Theorem 1 .**

The infection-free equilibrium *E*_00_ is globally asymptotically stable if and only if *R*_0_ < 1.

See Appendix A for proof.

**Theorem 2 .**

The equilibrium without immune response *E*_11_ is globally asymptotically stable if and only if *R*_1_ < 1.

See Appendix B for proof.

### 3.2. Local Asymptotic Stability and Hopf Bifurcation of the Positive Equilibrium

In this section, we will discuss the local asymptotic stability and Hopf bifurcation of the positive equilibrium *E*_22_.

The characteristic equation of system (1) at *E*_22_ is as follows:

(7) $$\begin{array}{c} H\left(\;\lambda ;\tau \;\right)=\left|\begin{array}{cccccc} \lambda +\rho +\beta v_{2} & 0 & 0 & \beta x_{2} & 0 & 0\\ -\beta v_{2} & \lambda +a_{1}+a_{2} & 0 & -\beta x_{2} & 0 & 0\\ 0 & -a_{2} & \lambda +a_{1}+k_{1}z_{2} & 0 & k_{1}y_{2} & 0\\ 0 & 0 & -k & \lambda +\varepsilon +k_{2}z_{2} & k_{2}v_{2} & 0\\ 0 & 0 & 0 & -k_{3}z_{2}e^{-\lambda \tau } & \lambda -k_{3}v_{2}e^{-\lambda \tau }+k_{4} & 0\\ 0 & 0 & -k_{1}k_{6}z_{2} & 0 & -k_{1}k_{6}y_{2} & \lambda +k_{7} \end{array}\right|=0. \end{array}$$

Define

(8) A1=2a1+a2+kz2,A2=a1+a2a1+kz2,A3=ε+k4−k2z2,A4=k4ε−k2z2,A5=a2kβx2,A6=a2kk4βx2,M1=−k3v2,M2=2k2z2−εk3v2,M3=kk1k3y2z2,M4=−a2kk3βx2v2+kk1k3y2z2a1+a2,s1=A1+A3,s2=A2+A4+A1A3,s3=A5+A1A4+A2A3,s4=A6+A2A4,s5=M1,s6=M2+A1M1,s7=A1M2+A2M1+M3,s8=A2M2+M4,s9=ρ+βv2,s10=β2x2v2a2k,s11=β2x2v2a2kk4,s12=−β2x2v22a2kk3,B1=s1+s9,B2=s2+s1s9,B3=s3+s2s9,B4=s4+s3s9+s10,B5=s11+s4s9,B6=s5,B7=s6+s5s9,B8=s7+s6s9,B9=s8+s7s9,B10=s12+s8s9,D1=B1+k7,D2=B2+B1k7,D3=B3+B2k7,D4=B4+B3k7,D5=B5+B4k7,D6=B6+B5k7,S1=B6,S2=B7+B6k7,S3=B8+B7k7,S4=B9+B8k7,S5=B10+B9k7,S6=B10k7.

Then the characteristic equation *H*(*λ*; *τ*) above becomes

(9) Hλ;τ=λ6+D1λ5+D2λ4+D3λ3+D4λ2+D5λ+D6+S1λ5e−λτ+S2λ4e−λτ+S3λ3e−λτ+S4λ2e−λτ+S5λe−λτ+S6e−λτ=0.

When *τ* = 0, (9) further becomes

(10) Hλ;τ=λ6+n1λ5+n2λ4+n3λ3+n4λ2+n5λ+n6=0,

where

(11) n1=D1+S1,n2=D2+S2,n3=D3+S3,n4=D4+S4,n5=D5+S5,n6=D6+S6.

Using the Routh-Hurwitz criterion [23], we obtain the following lemma.

**Lemma 3 .**

If (10) satisfies Δ_1_ ≡ *n*_1_ > 0,  $\Delta_{2}\equiv \left|\begin{array}{cc} n_{1} & 1\\ n_{3} & n_{2} \end{array}\right|>0$,  $\Delta_{3}\equiv \left|\begin{array}{ccc} n_{1} & 1 & 0\\ n_{3} & n_{2} & n_{1}\\ n_{5} & n_{4} & n_{3} \end{array}\right|>0$ and $\Delta_{4}=\left|\begin{array}{cccc} n_{1} & 1 & 0 & 0\\ n_{3} & n_{2} & n_{1} & 1\\ n_{5} & n_{4} & n_{3} & n_{2}\\ 0 & n_{6} & n_{5} & n_{4} \end{array}\right|>0$, then the positive equilibrium *E*_22_ is locally asymptotically stable when *τ* = 0.

**Proof**

By the Routh-Hurwitz criterion, if the four conditions are satisfied, then all roots of (10) have negative real parts. Therefore, the positive equilibrium *E*_22_ is locally asymptotically stable when *τ* = 0. For more details, we refer the readers to [25, 26].

From Lemma 3, we know that all roots of *H*(*λ*; *τ*) lie to the left of the imaginary axis when *τ* = 0. However, with *τ* increasing from zero, some of its roots may cross the imaginary axis to the right. In this case, there are some roots having positive real parts, and therefore the equilibrium *E*_22_ becomes unstable. Next, we will discuss the stability of system (1) at *E*_22_ when *τ* > 0.

We first divide (9) into two parts and obtain

(12) λ6+D1λ5+D2λ4+D3λ3+D4λ2+D5λ+D62=S1λ5+S2λ4+S3λ3+S4λ2+S5λ+S62e−λτ2.

Suppose (9) has a purely imaginary root *λ* = *iω*  (*ω* > 0). Substituting *λ* = *iω* into (12) yields

(13) −ω6+D1ω5i+D2ω4−D3ω3i−D4ω2+D5ωi+D62=S1ω5i+S2ω4−S3ω3i−S4ω2+S5ωi+S62.

By separating the real part and imaginary part, the following real part is obtained:

(14) ω12+C1ω10+C2ω8+C3ω6+C4ω4+C5ω2+C6=0,

where

(15) C1=−D12−2D2+S12,C2=D22+2D4+2D1D3−S22−2S1S3,C3=−D32−2D6−2D1D5−2D2D4+S32+2S1S5+2S2S4,C4=D42+2D2D6+2D3D5−S42−2S2S6−2S3S5,C5=−D52−2D4D6+S52+2S4S6,C6=D62−S62.

Let

(16) Gx=x6+C1x5+C2x4+C3x3+C4x2+C5x+C6.

Therefore, if (9) has a purely imaginary root *iω*, it is equivalent to the fact that *G*(*x*) = 0 has a positive real root *ω* ^2^.

**Theorem 4 .**

If *G*(*x*) = 0 has no positive real roots, then the positive equilibrium *E*_22_ is locally asymptotically stable for any *τ* > 0.

**Proof**

If *G*(*x*) = 0 has no positive real roots, then obviously (9) has no positive real roots. Therefore, the positive equilibrium *E*_22_ is locally asymptotically stable for any *τ* > 0.

Substituting *λ* = *iω* into (22), we obtain the real part,

(17) −ω6+D2ω4−D4ω2+D6+S1ω5−S3ω3+S5ωsin⁡ωτ+S2ω4−S4ω2+S6cos⁡ωτ=0,

and imaginary part,

(18) D1ω5−D3ω3+D5ω+−S2ω4+S4ω2−S6sin⁡ωτ+S1ω5−S3ω3+S5ωcos⁡ωτ=0.

Assuming that *G*(*x*) = 0 has $\tilde{n}(1\leq \tilde{n}\leq 6)$ positive real roots, denoted by $x_{n}\;\;(1\leq n\leq \tilde{n})$. As $\sqrt{x_{n}}=\omega$, we then have

(19) cos⁡xnτ=Qn=−S2xn2+S4xn−S6−xn3+D2xn2−D4xn+D6S2xn2−S4xn+S62+S1xn2xn−S3xnxn+S5xn2−S1xn2xn−S3xnxn+S5xnD1xn2xn−D3xnxn+D5xnS2xn2−S4xn+S62+S1xn2xn−S3xnxn+S5xn2,sin⁡xnτ=Pn=S2xn2−S4xn+S6D1xn2xn−D3xnxn+D5xnS2xn2−S4xn+S62+S1xn2xn−S3xnxn+S5xn2−S1xn2xn−S3xnxn+S5xn−xn3+D2xn2−D4xn+D6S2xn2−S4xn+S62+S1xn2xn−S3xnxn+S5xn2.

Let

(20) $$\begin{array}{c} \tau_{n}^{\left(j\right)}=\left\{\begin{array}{cc} \frac{1}{\sqrt{x_{n}}}\left[\arccos\left(Q_{n}\right)+2j\pi \right], & \text{if}\;\;P_{n}\geq 0,\\ \frac{1}{\sqrt{x_{n}}}\left[2\pi -\arccos\left(Q_{n}\right)+2j\pi \right], & \text{if}\;\;P_{n}<0, \end{array}\right. \end{array}$$

where $1\leq n\leq \tilde{n}$ and *j* = 0,1, 2,….

Therefore, the characteristic equation *H*(*λ*; *τ*_*n*_^(*j*)^) = 0 has a pair of purely imaginary roots $\pm i\sqrt{x_{n}}$. For every integer *j* and $1\leq n\leq \tilde{n}$, define *λ*_*n*_^(*j*)^(*τ*) = *α*_*n*_^(*j*)^(*τ*) + *iω*_*n*_^(*j*)^(*τ*) as the root of (9) near *τ*_*n*_^(*j*)^, satisfying *α*_*n*_^(*j*)^(*τ*_*n*_^(*j*)^) = 0 and $\omega_{n}^{\left(j\right)}(\tau_{n}^{\left(j\right)})=\sqrt{x_{n}}$. Then the following theorem is obtained.

**Theorem 5 .**

If *G*(*x*) = 0 has some positive real roots, then *E*_22_ is locally asymptotically stable for *τ* ∈ [0, *τ*_*n*_0__^(0)^), where

(21) $$\begin{array}{c} \tau_{n_{0}}^{\left(0\right)}=\min\left\{\;\tau_{n}^{\left(j\right)}\;|\;1\leq n\leq \tilde{n},\;\;j=0,1,2,\ldots \;\right\}. \end{array}$$

**Proof**

For $\tau_{n_{0}}^{\left(0\right)}=\min\left\{\tau_{n}^{\left(j\right)}|1\leq n\leq \tilde{n},\;\;j=0,1,2,\ldots \right\}$,  *G*(*x*) = 0 has no positive real roots when *τ* ∈ [0, *τ*_*n*_0__^(0)^), which means that all the roots of (9) have strictly negative real parts when *τ* ∈ [0, *τ*_*n*_0__^(0)^). Therefore, *E*_22_ is locally asymptotically stable for *τ* ∈ [0, *τ*_*n*_0__^(0)^).

**Theorem 6 .**

If *x*_*n*_0__ is a simple root of *G*(*x*) = 0, then there is a Hopf bifurcation for the system as *τ* increases past *τ*_*n*_0__^(0)^.

See Appendix C for proof.

## 4. Stability of the Bifurcating Periodic Solutions

In this section, we will continue to derive the explicit formulas for determining the stability, direction, and other properties of the Hopf bifurcation at a critical value *τ*_*n*_0__^(0)^ by means of the normal form and the center manifold theory [27].

First, we make the following hypotheses.

(1) Equation (9) has a pair of purely imaginary roots ±*iω*_0_ at *τ* = *τ*_0_, where $\tau_{0}\in \left\{\tau_{n}^{\left(j\right)}|1\leq n\leq \tilde{n},\;\;j=0,1,2,\ldots \right\}$.

(2) The remaining roots of (22) have strictly negative real parts.

(3) *ω*_0_ is a simple root of *G*(*x*) = 0.

We use *u* = *τ* − *τ*_0_ to represent a new bifurcation parameter. Let *X*(*t*) = (*x* − *x*_2_, *e* − *e*_2_, *y* − *y*_2_, *v* − *v*_2_, *z* − *z*_2_, *w* − *w*_2_)^*T*^, and *X*_*t*_(*θ*) = *X*(*t* + *θ*), where *θ* ∈ [−*τ*, 0]. Therefore, system (1) can be written as the following functional differential equation:

(22) $$\begin{array}{c} \dot{X}\left(\;t\;\right)=L_{u}X_{t}+f\left(\;X_{t}\left(\;\cdot \;\right),u\;\right), \end{array}$$

where

(23) Luϕ=F1ϕ0+F2ϕ−τ,F1=−ρ−βv200−βx200βv2−a1−a20βx2000a2−a1−k1z20−k1y2000k−ε−k2z2−k2v200000−k4000k1k6z20k1k6y2−k7,F2=000000000000000000000000000k3z2k3v20000000,fϕ,u=−βϕ10ϕ40βϕ10ϕ40−k1ϕ30ϕ50−k2ϕ40ϕ50k3ϕ4−τϕ5−τk1k6ϕ30ϕ50.

By the Riesz representation theorem [28], there exists a 6 × 6 matrix-valued function such that

(24) $$\begin{array}{c} L_{u}\phi =\overset{0}{\underset{-\tau }{\int }}d\eta \left(\;\theta ,u\;\right)\phi \left(\;\theta \;\right), \end{array}$$

where *dη*(*θ*, *u*) = *F*_1_ *δ*(*θ*)*dθ* + *F*_2_ *δ*(*θ* + *τ*)*dθ*.

For *ϕ* ∈ *C*([−*τ*, 0], *R* ^6^), we further define

(25) Auϕθ=dϕθdθ,if  θ∈−τ,0,∫−τ0dηξ,uϕξ≡Luϕ,if  θ=0,Ruϕθ=0,if θ∈−τ,0,fϕ,u,if θ=0.

Then system (22) can be written as

(26) $$\begin{array}{c} {\dot{X}}_{t}\left(\;\theta \;\right)=A\left(\;u\;\right)X_{t}\left(\;\theta \;\right)+R\left(\;u\;\right)X_{t}\left(\;\theta \;\right). \end{array}$$

For *φ* ∈ *C*([−*τ*, 0], *R* ^6^), define

(27) A∗0φs=dϕsds,if  s∈−τ,0,∫−τ0dηTξ,0φ−ξ,if  s=0,

and an inner product of *ϕ*, *φ*

(28) φ,ϕ=φ−T0ϕ0−∫θ=−τ0∫ξ=0θφ−Tξ−θdηθϕξdξ,

where *η*(*θ*) = *η*(*θ*, 0) and *ϕ* ∈ *C*([−*τ*, 0], *R* ^6^). Then *A*(0) and *A* ^*∗*^(0) are adjoint operators.

Let *h*(*θ*) and *h* ^*∗*^(*s*) be the eigenvectors of *A*(0) and *A* ^*∗*^(0) corresponding to the eigenvalues *iω*_0_ and −*iω*_0_, respectively. We choose *h*(*θ*) and *h* ^*∗*^(*s*) as

(29) hθ=1,h2,h3,h4,h5,h6Teiω0θ,h∗s=D1,h2∗,h3∗,h4∗,h5∗,h6∗Teiω0s,

so that 〈*h* ^*∗*^(*s*), *h*(*θ*)〉 = 1 is satisfied. We give the detailed computation of (29) in Appendix D.

In the following, we will compute the coefficients, *g*_20_,  *g*_11_,  *g*_02_, and *g*_21_, using the method given in [27]. The detailed computation of *g*_20_,  *g*_11_,  *g*_02_, and *g*_21_ is presented in Appendix E.

Then the following values can be computed:

(30) c10=i2ω0g11g20−2g112−g0223+g212,u2=−Re⁡c10Re⁡λ′τ0,β2=2Re⁡c10.

The signs of *u*_2_,  *β*_2_ determine the direction of the Hopf bifurcation and the stability of bifurcating periodic solutions, respectively [27]. From (C.11) of Appendix C, we obtain sign[(*dα*_*n*_^(*j*)^(*τ*)/*dτ*)|_*τ*=*τ*_*n*_^*j*^_] = sign[(*dG*/*dx*)|_*x*=*x*_*n*__].

Let *u*_2_^*∗*^ = −Re(*c*_1_(0))/*G*′(*ω*_0_^2^). We obtain the following theorem.

**Theorem 7 .**

Assume the hypotheses (1), (2), and (3) at the beginning of Section 4 hold.

(1) If *u*_2_^*∗*^ > 0  (*u*_2_^*∗*^ < 0), then the bifurcating periodic solutions exist for *τ* > *τ*_0_  (*τ* < *τ*_0_) in a *τ*_0_-neighborhood.

(2) If *β*_2_ < 0  (*β*_2_ > 0), the bifurcating periodic solutions are orbitally asymptotically stable as *t* → +*∞*  (*t* → −*∞*).

**Proof**

(1) If *τ*_0_ = *τ*_*n*_0__^(0)^, where $\tau_{n_{0}}^{\left(0\right)}=\min\left\{\tau_{n}^{\left(j\right)}|1\leq n\leq \tilde{n},\;\;j=0,1,2,\ldots \right\}$, and the hypotheses (1), (2), and (3) hold, then, from Theorem 6, we draw the conclusion that the existence and stability of the bifurcating periodic solutions are only determined by Re(*c*_1_(0)).

(2) If *β*_2_ < 0, namely, Re(*c*_1_(0)) < 0, then there exist stable periodic solutions for *τ* > *τ*_*n*_0__^(0)^ in a *τ*_0_-neighborhood. So the bifurcating periodic solutions are orbitally asymptotically stable as *t* → +*∞*.

## 5. Simulation and Discussions

In this section, we will numerically illustrate the theoretical results obtained above and also discuss how the factors such as the immune response to infected cells and viruses and the proliferation of hepatocytes affect the dynamics of the model under time delay.

For the following simulations, we choose the parameter values for system (1) as follows:

(31) λ=4.0551,r=0.6933,d=4.4096,β=4.6178,a1=0.0638,a2=1.8858,k1=0.8391,k=2.7011,ε=0.5083,k2=0.1963,k3=4.6661,k4=4.8580,k5=1.8046,k6=3.2210,k7=0.3397.

We set the initial values to *x*(*t*) = 1,  *e*(*t*) = 1,  *y*(*t*) = 1,  *v*(*t*) = 1,  *z*(*t*) = 1, and *w*(*t*) = 1, where *t* ∈ [−*τ*, 0].

### 5.1. Hopf Bifurcation and the Stability of Periodic Solutions

With the parameter values given in (31), we have the positive equilibrium *E*_22_ = (0.4757,1.1732,0.3281,1.0411,1.7470,9.8727) and the critical time value *τ*_*n*_0__^(0)^ = 0.041.

**Figure 2:**
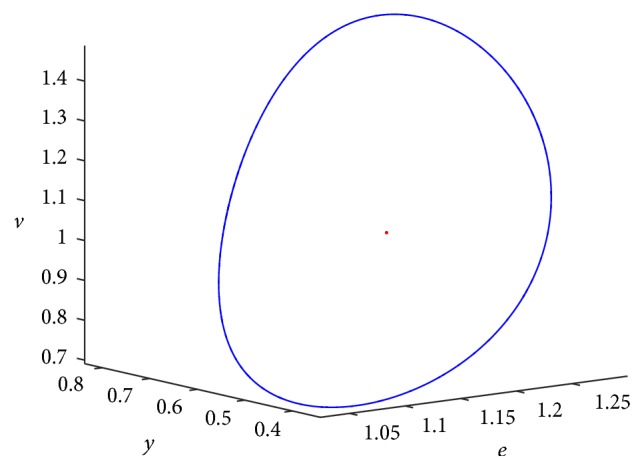
The positive equilibrium *E*_22_ bifurcates into a periodic solution at *τ* = 0.05 (a limit cycle).

**Figure 3:**
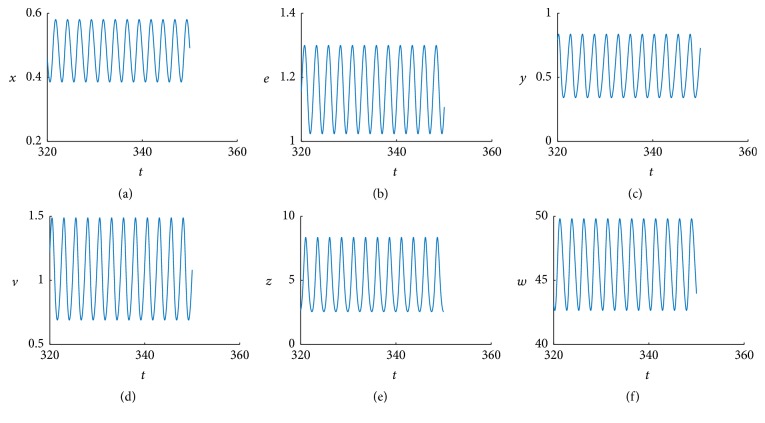
The positive equilibrium *E*_22_ bifurcates into a periodic solution at *τ* = 0.05.

When *τ* > 0.041, we obtain stable bifurcating periodic solutions. For example, when *τ* = 0.05, the simulation result is shown Figures 2 and 3.  Figure 2 indicates that a stable limit cycle is obtained as expected and Figure 3 indicates the state dynamics of uninfected cells, exposed cells, infected cells, free viruses, CTLs, and ALT, which are periodically oscillating.

**Figure 4:**
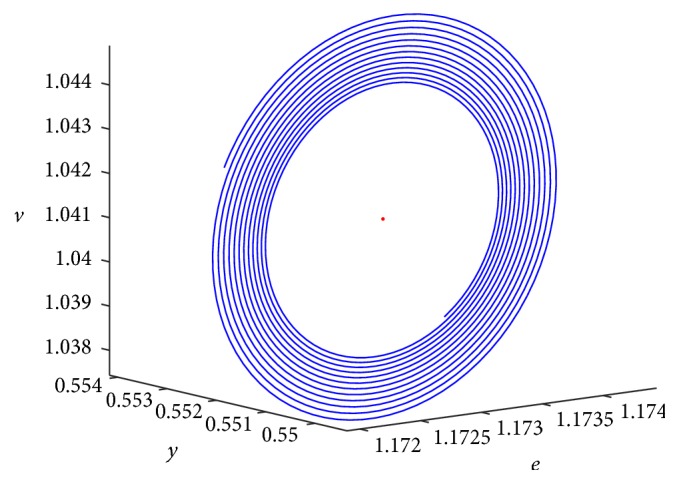
The positive equilibrium *E*_22_ at *τ* = 0.03.

**Figure 5:**
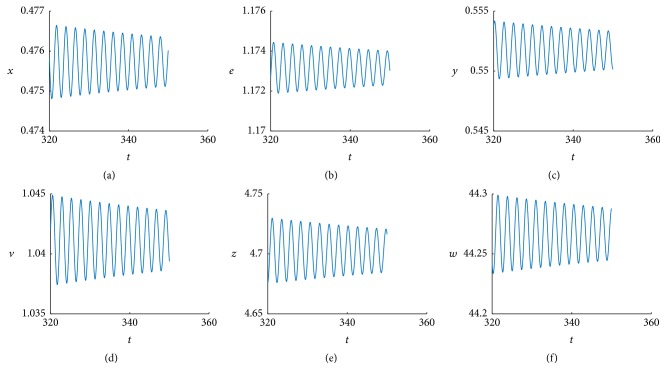
The positive equilibrium *E*_22_ remains stale at *τ* = 0.03.

When *τ* < 0.041, the bifurcating periodic solutions are unstable. For example, when *τ* = 0.03, the simulation result is shown in Figures 4 and 5. From Figures 4 and 5, we know that the positive equilibrium *E*_22_ is asymptotically stable and the system will converge to *E*_22_.

**Figure 6:**
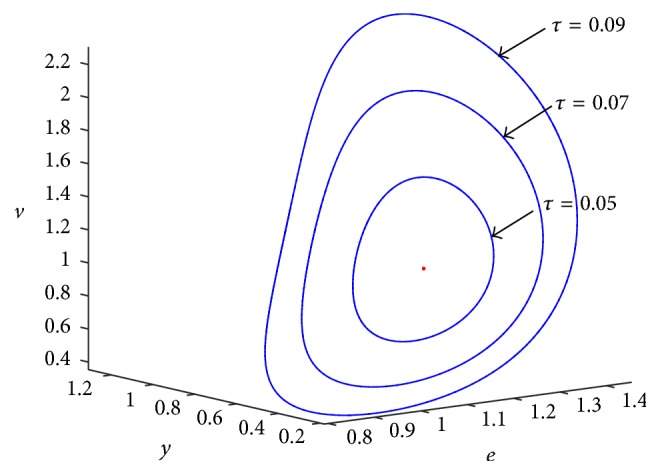
The different periodic solutions at *τ* = 0.05, *τ* = 0.07 and *τ* = 0.09 (three limit cycles).

With the increasing of time delay (*τ*), the radius of limit cycle will increase. The simulation result is shown Figure 6.

### 5.2. The Immune Response to Infected Cells

**Figure 7:**
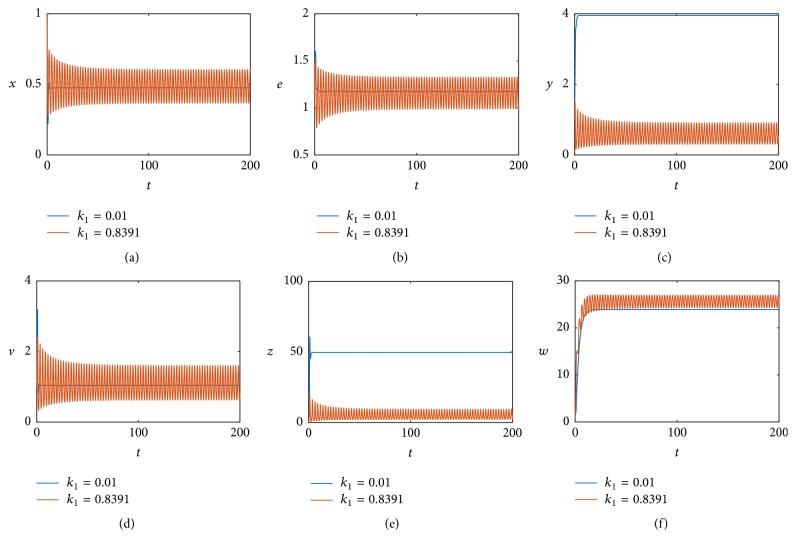
The positive equilibrium *E*_22_ remains stable at *τ* = 0.05 when *k*_1_ = 0.01 (blue line) and exists at stable periodic solutions when *k*_1_ = 0.8391 (red line).

Here we will investigate the effect of the immune response to infected cells on the model dynamics under time delay. When we change *k*_1_ = 0.8391 to *k*_1_ = 0.01 by fixing other values given in (31), we obtain a simulation result at *τ* = 0.05, illustrated in Figure 7. Comparing Figure 2 when *k*_1_ = 0.8391 and Figure 7 when *k*_1_ = 0.01, we can see that, with the decrease of *k*_1_, the stable periodic solution becomes unstable, that is, asymptotically stable.

### 5.3. The Immune Response to Viruses

**Figure 8:**
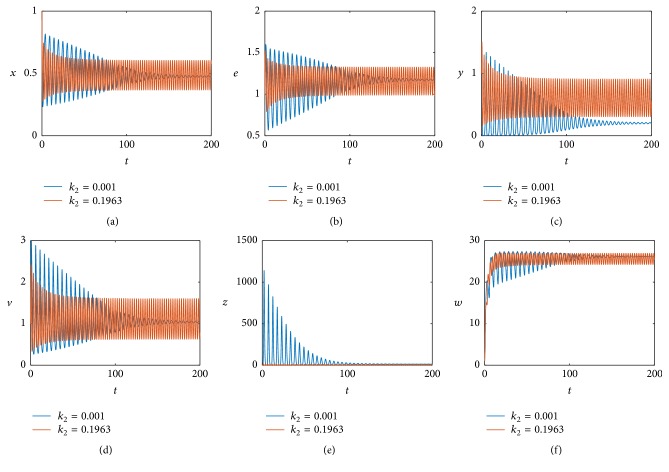
The positive equilibrium *E*_22_ remains stable at *τ* = 0.05 when *k*_2_ = 0.001 (blue line) and exists at stable periodic solutions when *k*_2_ = 0.1963 (red line).

We continue to investigate the effect of the immune response to viruses on the model dynamics under time delay. When we change *k*_2_ = 0.1963 to *k*_2_ = 0.001 by fixing other values given in (31), we obtain a simulation result at *τ* = 0.05, illustrated in Figure 8. Similarly, comparing Figures 2 and 8, we can see that, with the decrease of *k*_2_, the stable periodic solution also becomes unstable, that is, asymptotically stable. We further can see that the effect of the immune response to infected cells on the model dynamics is similar to that of the immune response to viruses.

### 5.4. Proliferation of Hepatocytes

**Figure 9:**
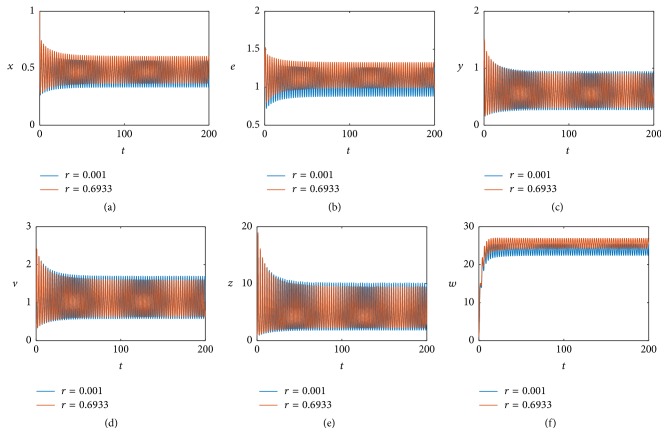
The positive equilibrium *E*_22_ remains stable at *τ* = 0.05 when *r* = 0.001 (blue line) and exists at stable periodic solutions when *r* = 0.6933 (red line).

Then we investigate the effect of proliferation of hepatocytes on the model dynamics under time delay. For this, we still keep *τ* = 0.05 and change the value of parameter *r*. When we change *r* = 0.6933 to *r* = 0.001 by fixing other values given in (31), we obtain a simulation result at *τ* = 0.05, illustrated in Figure 9. Figure 9 shows that when *r* = 0.001, the bifurcating periodic solution is stable, compared with Figure 2 when *r* = 0.6933. We also try other values of parameter rand obtaining similar results. Thus, we can see parameter *r* has a small effect on the model dynamics, which reflect in periodicity and the positive equilibrium *E*_22_.

## 6. Conclusions

In this paper, we consider a comprehensive delayed HBV model. Different from other existing models, our model not only considers the immune response to both infected cells and viruses and a time delay for the immune system to clear viruses but also incorporates an exposed state and the proliferation of hepatocytes.

We then prove the positivity and boundedness of solutions and analyze the global stability of two boundary equilibria and investigate the local asymptotic stability and Hopf bifurcation of the positive (infection) equilibrium and also the stability of the bifurcating periodic solutions. We also numerically illustrate the Hopf bifurcation and the stability of the bifurcating periodic solutions.

Moreover, we numerically illustrate how the factors such as the time delay, the immune response to infected cells and viruses, and the proliferation of hepatocytes affect the dynamics of the model, which shows that the former two factors have a big effect on the model dynamics, while the latter one does not have a big effect.

## Appendix

### A. The Proof of Theorem 1

We construct a Lyapunov functional as follows:

(A.1) V0=a2ka1+a2xt−x0−x0ln⁡xtx0+a2ka1+a2et+kyt+a1vt.

Calculating the derivative of *V*_0_ along with the trajectories of system (1), we obtain

(A.2) V˙0=a2ka1+a21−x0xtλ−ρx−βxv+a2ka1+a2βxv−a1et−a2et+ka2et−a1y−k1yz+a1ky−εv−k2vz=−a2kρa1+a2·xt−x02xt−a2ka1+a2βxv+a2ka1+a2·x0xt·βxv+a2ka1+a2βxv−a2ka1+a2a1+a2et+ka2et−ka1y−kk1yz+a1ky−a1εv−a1k2vz=−a2kρa1+a2·xt−x02xt−a1ε1−a2kx0βa1εa1+a2v−kk1yz−a1k2vz=−a2kρa1+a2·xt−x02xt−a1ε1−R0v−kk1yz−a1k2vz.

Therefore, when *R*_0_ = *a*_2_ *kx*_0_ *β*/*a*_1_ *ε*(*a*_1_ + *a*_2_) < 1,  ${\dot{V}}_{0}\leq 0$ holds true. Furthermore, if and only if *x* = *x*_0_, *e* = 0, *y* = 0, *v* = 0, *z* = 0, and *w* = *w*_0_, the Lyapunov functional satisfies ${\dot{V}}_{0}=0$. According to LaSalle's invariance principle [29], the infection-free equilibrium *E*_00_ is globally asymptotically stable when *R*_0_ < 1. Apparently, when *R*_0_ > 1,  *E*_00_ is unstable.

### B. The Proof of Theorem 2

Similarly, we define the following Lyapunov functional:

(B.1) V1=k3εβx1xt−x1−x1ln⁡xtx1+a2kk3a1a1+a2et−e1−e1ln⁡ete1+kk3a1yt−y1−y1ln⁡yty1+k3vt−v1−v1ln⁡vtv1+k2z+k2k3∫t−τtvθzθdθ.

The derivative of *V*_1_ along with the trajectories of system (1) can be calculated as

(B.2) V˙1=k3εβx11−x1xλ−ρx−βxv+a2kk3a1a1+a21−e1et·βxv−a1et−a2et+kk3a11−y1y·a2et−a1y−k1yz+k31−v1v·ky−εv−k2vz+k2k3vt−τzt−τ−k4z+k2k3∫t−τtvθzθdθ=−k3εβx1ρx1−x2x+k3εβx1βx1v1−k3εβx1βxv−m1βx12v1x+k3εβx1βx1v+a2kk3a1a1+a2βxv−a2kk3a1a1+a2a1et−a2kk3a1a1+a2a2et−a2kk3a1a1+a2e1etβxv+a2kk3a1a1+a2a1e1+a2kk3a1a1+a2a2e1+kk3a1·a2et−kk3a1a1y−kk3a1k1yz−kk3a1y1a2ety+kk3a1y1a1+kk3a1y1k1z+k3ky−k3εv−k3k2vz−k3v1kyv+k3v1ε+k3v1k2z+k2k3vt−τ·zt−τ−k2k4z+k2k3vz−k2k3vt−τ·zt−τ=−k3εβx1ρx1−x2x+k3εv14−x1x−y1ete1y−xe1vx1etv1−v1yy1v+k3v1k2−k2k4+kk3a1y1k1z−kk3a1k1yz=−a2kk3a1a1+a2ρx1−x2x+k3εv14−x1x−y1ete1y−xe1vx1etv1−v1yy1v−k2k41−R1z−kk3a1k1yz.

Therefore, *R*_1_ = *k*_3_ *v*_1_/*k*_4_ + *kk*_3_ *y*_1_ *k*_1_/*a*_1_ *k*_2_ *k*_4_ < 1 ensures that ${\dot{V}}_{0}\leq 0$ holds true. Furthermore, if and only if *x* = *x*_1_,  *e* = *e*_1_,  *y* = *y*_1_,  *v* = *v*_1_,  *z* = 0, and *w* = *w*_1_, the Lyapunov functional satisfies ${\dot{V}}_{0}=0$. Using the LaSalle's invariance principle, we can see that the equilibrium without immune response *E*_11_ = (*x*_1_, *e*_1_, *y*_1_, *v*_1_, 0, *w*_1_) is globally asymptotically stable.

### C. The Proof of Theorem 6

The characteristic equation (9) can be written into the following form:

(C.1) $$\begin{array}{c} f_{0}\left(\;\lambda \;\right)+f_{1}\left(\;\lambda \;\right)e^{-\lambda \tau }=0, \end{array}$$

where

(C.2) f0λ=λ6+D1λ5+D2λ4+D3λ3+D4λ2+D5λ+D6,f1λ=S1λ5+S2λ4+S3λ3+S4λ2+S5λ+S6,

and *f*_0_(*λ*) and *f*_1_(*λ*) are continuously differentiable to *λ*.

Suppose that one of the roots of (C.1) is *λ*(*τ*) = *α*(*τ*) + *iω*(*τ*), satisfying *α*(*τ*_0_) = 0 and *ω*(*τ*_0_) = *ω*_0_ for a positive real number *τ*_0_.

Let

(C.3) $$\begin{array}{c} \Phi \left(\;\omega \;\right)={\left|\;f_{0}\left(\;i\omega \;\right)\;\right|}^{2}-{\left|\;f_{1}\left(\;i\omega \;\right)\;\right|}^{2}. \end{array}$$

Calculating the derivative of |*f*_0_(*iω*)|^2^ to *ω*, we have

(C.4) ddωf0iω2=−2 lm⁡f0iω¯f˙0iω=12ω11+−20D2+10D12ω9+16D4−16D1D3+8D22ω7+12D1D5−12D2D4+6D32−12D6ω5+−8D3D5+4D42+8D2D6ω3+2D52−4D4D6ω.

Then we have

(C.5) 12ω·dΦdω=12ω·ddωf0iω2−f1iω2=lm⁡f1iω2f˙1iωωf1iω−f0iω2f˙0iωωf0iω.

Because |*f*_0_(*iω*_0_)|^2^ = |*f*_1_(*iω*_0_)|^2^, we have

(C.6) 12ω·dΦdωω=ω0=f0iω02lm⁡f˙1iω0ω0f1iω0−f˙0iω0ω0f0iω0.

Calculating the derivative of both sides of (C.1) to *τ*, we have

(C.7) f˙0λdλdτ+f˙1λdλdτe−λτ−λ+τdλdτf1λe−λτ=0.

Then we have

(C.8) dλdτ−1=f˙0λ+f˙1λe−λτ−τf1λe−λτλf1λe−λτ=f˙0λeλτ+f˙1λλf1λ−τλ.

Since *f*_0_(*iω*_0_) + *f*_1_(*iω*_0_)*e* ^−*iω*_0_*τ*^ = 0, we obtain

(C.9) Re⁡dsdττ=τ0−1=Re⁡f˙0iω0eiω0τ+f˙1iω0iω0f1iω0=Re⁡f˙0iω0ω0f0iω0i+Re⁡−f˙1iω0ω0f1iω0i=lm⁡f˙1iω0ω0f1iω0−f˙0iω0ω0f0iω0.

Thus, we have

(C.10) sign⁡dRe⁡λdττ=τ0=sign Re⁡dλdττ=τ0=sign Re⁡dλdττ=τ0−1=sign⁡12ω·dΦdωω=ω0.

When Re⁡(*λ*) = *α*_*n*_^(*j*)^(*τ*), obviously, we have

(C.11) $$\begin{array}{c} \mathrm{sign}\left[\;{\left.\;\frac{d\alpha_{n}^{\left(j\right)}\left(\tau \right)}{d\tau }\;\right|}_{\tau =\tau_{n}^{j}}\;\right]=\mathrm{sign}\left[\;{\left.\;\left(\;\frac{dG}{dx}\;\right)\;\right|}_{x=x_{n}}\;\right]. \end{array}$$

As *x*_*n*_0__ is a simple root of *G*(*x*) = 0, we know $\dot{G}(x_{n_{0}})\neq 0$. From (C.11), we further know (*dα*_*n*_0__^(0)^/*dτ*)|_*τ*=*τ*_*n*_0__^(0)^_ ≠ 0. If (*dα*_*n*_0__^(0)^/*dτ*)|_*τ*=*τ*_*n*_0__^(0)^_ < 0; we obtain that the roots of (9) have positive real part when *τ* ∈ [0, *τ*_*n*_0__^(0)^), which contrasted with Theorem 5. Hence, we can see that (*dα*_*n*_0__^(0)^/*dτ*)|_*τ*=*τ*_*n*_0__^(0)^_ > 0. When *τ* = *τ*_*n*_0__^(0)^, except for the pair of purely imaginary roots, the remaining roots of *H*(*λ*; *τ*) have strictly negative real parts, so the system has Hopf bifurcation.

### D. The Detailed Computation of (29)

In what follows, we will compute (29).

Substituting *X* = *e* ^*λt*^ into (26), we have

(D.1) λeλt=F1eλt+F2eλte−λt⟹λI−F1−F2e−λteλt=0.

Therefore, det|*λI* − *F*_1_ − *F*_2_ *e* ^−*λt*^| = 0 is the characteristic equation of *A*(0).

As *A*(0) = *F*_1_ + *F*_2_ *e* ^−*iω*_0_*τ*_0_^, we have

(D.2) $$\begin{array}{c} \left(\;A\left(\;0\;\right)-i\omega_{0}I\;\right)h\left(\;\theta \;\right)=0, \end{array}$$

where

(D.3) $$\begin{array}{c} A\left(\;0\;\right)=\left[\begin{array}{cccccc} -\rho -\beta v_{2} & 0 & 0 & -\beta x_{2} & 0 & 0\\ \beta v_{2} & -a_{1}-a_{2} & 0 & \beta x_{2} & 0 & 0\\ 0 & a_{2} & -a_{1}-k_{1}z_{2} & 0 & -k_{1}y_{2} & 0\\ 0 & 0 & k & -\varepsilon -k_{2}z_{2} & -k_{2}v_{2} & 0\\ 0 & 0 & 0 & k_{3}z_{2}e^{-i\omega_{0}\tau_{0}} & -k_{4}+k_{3}v_{2}e^{-i\omega_{0}\tau_{0}} & 0\\ 0 & 0 & k_{1}k_{6}z_{2} & 0 & k_{1}k_{6}y_{2} & -k_{7} \end{array}\right]. \end{array}$$

So we have

(D.4)

The eigenvectors of *A*(0) are

(D.5) h1=1,h2=−ρ−iω0a1+a2+iω0,h3=a2−ρ−iω0a1+k1z2+iω0a1+a2+iω0−k1y2h5k3z2e−iω0τ0−ρ−βv2−iω0a1+k1z2+iω0βx2k4−k3z2e−iω0τ0+iω0,h4=−ρ−βv2−iω0βx2,h5=k3z2e−iω0τ0−ρ−βv2−iω0βx2k4−k3z2e−iω0τ0+iω0,h6=k1k6z2k7+iω0a2−ρ−iω0a1+k1z2+iω0a1+a2+iω0−k1y2h5k3z2e−iω0τ0−ρ−βv2−iω0a1+k1z2+iω0βx2k4−k3z2e−iω0τ0+iω0+k1k6y2k3z2e−iω0τ0−ρ−βv2−iω0k7+iω0βx2k4−k3z2e−iω0τ0+iω0.

Similarly,

(D.6) A∗0=−ρ−βv2βv200000−a1−a2a200000−a1−k1z2k0k1k6z2−βx2βx20−ε−k2z2k3z2e−iω0τ0000−k1y2−k2v2−k4+k3v2e−iω0τ0k1k6y200000−k7−ρ−βv2+iω0βv200000−a1−a2+iω0a200000−a1−k1z2+iω0k0k1k6z2−βx2βx20−ε−k2z2+iω0k3z2eiω0τ0000−k1y2−k2v2−k4+k3v2eiω0τ0+iω0k1k6y200000−k7+iω01h2∗h3∗h4∗h5∗h6∗=0.

The eigenvectors of *A* ^*∗*^(0) are

(D.7) h1∗=1,h2∗=−−ρ−βv2+iω0βv2,h3∗=−a1+a2−iω0−ρ−βv2+iω0a2βv2,h4∗=−a1+k1z2−iω0a1+a2−iω0−ρ−βv2+iω0a2kβv2,h5∗=−k1y2a1+a2−iω0−ρ−βv2+iω0a2βv2−k4+k3v2eiω0τ0+iω0−k2a1+k1z2−iω0a1+a2−iω0−ρ−βv2+iω0a2kβ−k4+k3v2eiω0τ0+iω0,h6∗=0.

Since

(D.8) 1=h∗,h=D−1,h−2∗,h−3∗,h−4∗,h−5∗,h−6∗1h2h3h4h5h6−∫−τ00∫ξ=0θD−1,h−2∗,h−3∗,h−4∗,h−5∗,h−6∗·e−iω0ξ−θdηθ1h2h3h4h5h6eiω0ξdξ=D−1+h2h−2∗+h3h−3∗+h4h−4∗+h5h−5∗+h6h−6∗−limn→∞⁡D−1,h−2∗,h−3∗,h−4∗,h−5∗,h−6∗∑i=1neiω0ξiθiηθi−ηθi−11h2h3h4h5h6=D−1+h2h−2∗+h3h−3∗+h4h−4∗+h5h−5∗+h6h−6∗−τ0e−iω0τ0k3zh4h5∗−τ0e−iω0τ0k3vh5h5∗,

we have that

(D.9) D=1+h2h−2∗+h3h−3∗+h4h−4∗+h5h−5∗+h6h−6∗−τ0e−iω0τ0k3zh4h5∗−τ0e−iω0τ0k3vh5h5∗−1.

### E. The Detailed Computation of *g*_20_, *g*_11_, *g*_02_, and *g*_21_

In the following, we will compute coefficients, *g*_20_,  *g*_11_,  *g*_02_, and *g*_21_, using the method given in [27].

Let

(E.1) fϕ,0=−βϕ10ϕ40βϕ10ϕ40−k1ϕ30ϕ50−k2ϕ40ϕ50k3ϕ4−τϕ5−τk1k6ϕ30ϕ50=fz2z22+fzz−zz−+fz−2z−22+fz2z−z2z−2,Xt=ϕ1ϕ2ϕ3ϕ4ϕ5ϕ6=zhθ+z−h−θ+wz,z−,θ=z1h2h3h4h5h6eiω0θ+z−1h2∗h3∗h4∗h5∗h6∗eiω0θ+wz,z−,θ,

where

(E.2) wz,z−,θ=w20θz22+w11θzz−+w02θz−22+⋯.

Then we have

(E.3) ϕ10=z+z−+w200z22+w110zz−+w020z−22+⋯,ϕ30=zh3+z−h−3+w2030z22+w1130zz−+w0230z−22+⋯,ϕ40=zh4+z−h−4+w2040z22+w1140zz−+w0240z−22+⋯,ϕ50=zh5+z−h−5+w2050z22+w1150zz−+w0250z−22+⋯,ϕ4−τ=zh4e−iω0τ+z−h−4eiω0τ+w204−τz22+w114−τzz−+w024−τz−22+⋯,ϕ5−τ=zh5e−iω0τ+z−h−5eiω0τ+w205−τz22+w115−τzz−+w025−τz−22+⋯,βϕ10ϕ40=2βh4z22+βh4+h−4zz−+2βh−4z−22+βw200h−4+w2020+2w1120+2h4w110z2z−2+⋯,−k1ϕ30ϕ50=−2k1h3h5z22−k1h3h−5+h−3h5·zz−−2k1h−3h−5z−22−k12h3w1150+h−3w205+h−5w2040+2h5w1140z2z−2+⋯,k1k6ϕ30ϕ50=2k1k6h3h5z22+k1k6h3h−5+h−3h5zz−+2k1k6h−3h−5z−22+k1k62h3w1150+h−3w205+h−5w2040+2h5w1140z2z−2+⋯,−k2ϕ40ϕ50=−2k2h4h5z22−k2h4h−5+h−4h5·zz−−2k2h−4h−5z−22−k22h4w1150+h−4w205+h−5w2040+2h5w1140z2z−2+⋯,k3ϕ4−τϕ5−τ=2k3h4h5e−2iω0τz22+k3h4h−5+h−4h5zz−+2k3h−4h−5e2iω0τz−22+k32h4w115−τe−iω0τ+h−4w205−τeiω0τ+h−5w204−τeiω0τ+2h5w114−τe−iω0τz2z−2+⋯.

We further obtain

(E.4) fϕ,0=−2βh42βh4−2k1h3h5−2k2h4h52k3h4h5e−2iω0τ2k1k6h3h5z22+−βh4+h−4βh4+h−4−k1h3h−5+h−3h5−k2h4h−5+h−4h5k3h4h−5+h−4h5−k1k6h3h−5+h−3h5zz−+−2βh−42βh−4−2k1h−3h−5−2k2h−4h−52k3h−4h−5e2iω0τk1k6h−3h−5z−22+−βw200h−4+w2020+2w1120+2h4w110βw200h−4+w2020+2w1120+2h4w110−k12h3w1150+h−3w205+h−5w2040+2h5w1140−k22h4w1150+h−4w205+h−5w2040+2h5w1140k32h4w115−τe−iω0τ+h−4w205−τeiω0τ+h−5w204−τeiω0τ+2h5w114−τe−iω0τk1k62h3w1150+h−3w205+h−5w2040+2h5w1140z2z−2.

Using the method [27], we obtain the following coefficients:

(E.5) g20=h−∗0fz2=D−1,h−2∗,h−3∗,h−4∗,h−5∗,h−6∗−2βh42βh4−2k1h3h5−2k2h4h52k3h4h5e−2iω0τ2k1k6h3h5,g11=h−∗0fzz−=D−1,h−2∗,h−3∗,h−4∗,h−5∗,h−6∗−βh4+h−4βh4+h−4−k1h3h−5+h−3h5−k2h4h−5+h−4h5k3h4h−5+h−4h5−k1k6h3h−5+h−3h5,g02=h−∗0fz−2=D−1,h−2∗,h−3∗,h−4∗,h−5∗,h−6∗−2βh−42βh−4−2k1h−3h−5−2k2h−4h−52k3h−4h−5e2iω0τk1k6h−3h−5,g21=h−∗0fz2z−=D−1,h−2∗,h−3∗,h−4∗,h−5∗,h−6∗·−βw200h−4+w2020+2w1120+2h4w110βw200h−4+w2020+2w1120+2h4w110−k12h3w1150+h−3w205+h−5w2040+2h5w1140−k22h4w1150+h−4w205+h−5w2040+2h5w1140k32h4w115−τe−iω0τ+h−4w205−τeiω0τ+h−5w204−τeiω0τ+2h5w114−τe−iω0τk1k62h3w1150+h−3w205+h−5w2040+2h5w1140,

where

(E.6) w20θ=ig20ω0eiω0θh0+ig−203ω0e−iω0θh−0+E20e2iω0θ,w11θ=−ig11ω0eiω0θh0+ig−11ω0e−iω0θh−0+E11,

where

(E.7) E20=2iω0I−∫−τ00e−2iω0θdη0,θ−1fz2=2iω0I−limλ→0⁡e2iω0ξ1ηθ1−ηθ0+⋯+e2iω0ξnηθn−ηθn−1−1fz2=2iω0I−limλ→0⁡e2iω0ξ1F2+e2iω0ξnF1−1fz2=2iω0I−F1−F2e−2iω0τ−1fz2=2iω0I−F1−F2e−2iω0τ−1·−2βh42βh4−2k1h3h5−2k2h4h52k3h4h5e−2iω0τ2k1k6h3h5,E11=−∫−τ00dη0,θ−1·fzz−=−F1+F2−1·fzz−=−2F1+F2−1−βRe⁡h4βRe⁡h4−k1Re⁡h3h−5−k2Re⁡h4h−5k3Re⁡h4h−5−k1k6Re⁡h3h−5.

So far, we have obtained *g*_20_,  *g*_11_,  *g*_02_, and *g*_21_.
